# Variation in Functional Independence among Stroke Survivors Having Fatigue and Depression

**DOI:** 10.1155/2013/842980

**Published:** 2013-09-11

**Authors:** Umaru Muhammad Badaru, Omoyemi Olubunmi Ogwumike, Ade Fatai Adeniyi, Olajide Olubanji Olowe

**Affiliations:** ^1^Department of Physiotherapy, Faculty of Medicine, Bayero University, Kano 700241, Nigeria; ^2^Department of Physiotherapy, College of Medicine, University of Ibadan, Ibadan 200211, Nigeria; ^3^National Orthopaedic Hospital Dala, Kano 700252, Nigeria

## Abstract

*Objective*. This study evaluated variation in functional independence in activities of daily living (ADL) and instrumental activities of daily living (IADL) among individuals with poststroke fatigue (PSF) and poststroke depression (PSD). *Methods*. A cross-sectional survey involved 65 consenting poststroke survivors who were purposively recruited from physiotherapy clinics of the University College Hospital, Ibadan, Adeoyo Maternity Teaching Hospital, Ibadan, and Federal Medical Center, Gusau. Participants were assessed for symptoms of PSD with short geriatric depression scale-15, PSF with fatigue severity scale, ADL with Barthel Index and IADL with Nottingham extended ADL scale. Data analysis was done using Chi-square and unpaired *t*-test with significance level being 0.05. *Results*. Participants' age ranged from 58 to 80 years. PSD alone (*P* = 0.002) and both PSF and PSD (*P* = 0.02) were significantly associated with ADL, while PSF alone was not (*P* = 0.233). PSD alone (*P* = 0.001) and both PSF and PSD (*P* = 0.001) significantly negatively affected IADL, while PSF alone had no significant effect (*P* = 0.2). *Conclusions*. Participants with PSD alone and those with both PSF and PSD had lower functional independence in ADL and IADL.

## 1. Introduction

In rehabilitation, assessment of functional independence is used to identify disabilities in activities of daily living (ADL). ADL is considered a primary functional status measure in stroke rehabilitation because of their relative objectivity, simplicity, and relevance to patients [1]. ADL include feeding, grooming, dressing, bathing, toileting, and transfers, while instrumental activities of daily living (IADL) comprise mobility, hand function, and social participation [2]. 

Stroke imposes serious restrictions on the ability to activate, use, and restore physiologic and psychosocial resources, thus promoting imbalance that results in subjective fatigue [3]. Fatigue has a debilitating influence on ADL [4, 5] and is independently associated with health related quality of life. Poststroke fatigue can be defined as a subjective experience and includes such symptoms as rapid inanition, persisting lack of energy, exhaustion, physical and mental tiredness, and apathy [6]. Poststroke fatigue has been attributed to functional impairment after stroke, and its recognition and treatment are important for maximizing recovery [7].

Depression after stroke is common and the effect of poststroke fatigue and depression on functional recovery after stroke has been documented in the literature. Depressed stroke patients have been found to be 30% less likely than nondepressed patients to achieve ADL and 40% less likely to be independent in three or more IADL [8]. Poststroke depression and severity of stroke are among other variables to predict functional independence of stroke survivors at discharge [9]. Nannetti et al. [10] observed that poststroke depression has a negative impact on functional recovery process after discharge but not during hospitalization. 

Many investigators have observed sex differences in stroke presentation and recovery [11–14]. According to Lai et al. [12], female stroke patients were less likely than male stroke patients to achieve basic ADL independence (Barthel Index of ≥95) and less likely than males to achieve complete independence in eight of the nine IADL 6 months after stroke. Poststroke depression status after stroke was one of the named contributing factors for weakness in women [12]. Although poststroke depression disorders have been observed to be higher in females than in males [13] and females were twice as frequently diagnosed with major poststroke depression than males [14], it has been found that major poststroke depression in men was associated with greater impairment in ADL [14].

Most of the aforementioned studies have shown that either poststroke fatigue alone or poststroke depression alone can significantly affect functional independence in ADL and IADL in both males and females. Some authors have also opined that poststroke fatigue may be accompanied by poststroke depression [5, 7, 15] and this has great impact on functional abilities. It has been observed that poststroke fatigue accompanying poststroke depression is often relieved when the depression is adequately treated, and that less is known about the occurrence of poststroke fatigue in the absence of poststroke depression [15]. A suggestion consequent to a study by van de Port et al. [5] is that when examining the impact of poststroke fatigue on functional abilities, poststroke depression may be a confounder which needs to be controlled. This present study is consequently set out to investigate whether poststroke fatigue could exist in the absence of poststroke depression and to investigate the influence of fatigue alone, depression alone, and the influence of both fatigue and depression on functional independence in ADL and IADL after stroke. This study specifically aimed at evaluating the differences in functional independence in ADL and IADL among stroke patients who have poststroke fatigue and poststroke depression symptoms together and exclusively.

## 2. Methodology

This research was a cross-sectional survey involving 65 consenting participants who were recruited from out-patient physiotherapy clinics of University College Hospital, Ibadan, Adeoyo Maternity Teaching Hospital, Ibadan, and Federal Medical Center, Gusau, all in Nigeria. Participants were purposively recruited based on the following inclusion criteria: they were all discharged from in-patient care, all presented with a first ever stroke, and all were not diagnosed with comorbidities that could significantly affect functional recovery in ADL and IADL. Such comorbidities were severe heart disease, severe knee and hip joint osteoarthritis, severe rheumatoid arthritis, joint deformities, severe peripheral diabetic neuropathy, visual impairment, severe obesity, aphasia, and dementia. Past medical history was taken to exclude participants with prestroke history of psychiatric disorder and any muscle weakness not directly associated with stroke. The procedure for data collection involved explaining the aims of the research to each participant after which the consent for participation was sought and obtained. 

Symptoms of poststroke depression were assessed with short geriatric depression scale-15 (GDS-15), poststroke fatigue with fatigue severity scale (FSS), instrumental activities of daily living with Nottingham extended ADL Scale, and Activities of Daily Living with Barthel Index (BI). The participants were directly assessed for ADL while they were asked about their IADLs. All the instruments were completed by the researchers. The IADL abilities were verified only when a patient reported high IADL but scored less than 100% on direct assessment of ADL. All the participants were blinded to the Nottingham extended ADL scale. 

### 2.1. Assessment of Poststroke Depression

Poststroke depression was assessed with the short geriatric depression scale-15 (GDS-15). It was a 15-item questionnaire scored on a 2-point scale. The minimum score for the GDS-15 was 0 and the maximum score was 15.0—4 points were considered as no depression, 5–9 points mild depression, and 10–15 points moderate to severe depression [16]. The reliability coefficient of GDS-15 was 0.81 [17] and the interrater reliability was 0.85 [18]. Its sensitivity ranges from 79% to 100% and specificity between 67% and 80% [19, 20]. The score of poststroke depression was dichotomized for the purpose of analysis into depressed (score > 4 points) and not depressed (score ≤ 4 points). 

### 2.2. Assessment of Poststroke Fatigue

Poststroke fatigue was assessed using the fatigue severity scale (FSS). It is consisted of 9 items that were rated by degree of agreement across a 7-point scale. The scores of each item ranged from 1 to 7. The total score of the FSS was the mean of the 9 items. The lowest mean score was 1 and the highest mean score was 7. Participants with a mean of 4 or more were considered having fatigue [21]. FSS has internal consistency of *α* = 0.89 [22].

### 2.3. Assessment of Activities of Daily Living (ADL)

The instrument used for the assessment of ADL was the Barthel Index (BI). It contains a total of 10 items describing different activities. The scoring is done by adding individual item score to give a total score ranging from 0 (totally dependent) to 100 (completely independent). Lower scores indicate greater dependency. Scoring on the BI is interpreted as 80–100 independent, 60–79 needs minimal help with ADL, 40–59 partially dependent, 20–39 very dependent, and ascore < 20, totally dependent [23]. The score of 80–100 was therefore considered independent and a score <80 was considered dependent. The BI has good internal consistency with a Cronbach's alpha coefficient of 0.98; intrarater and inter-rater reliabilities are high, with a Pearson's *r* score ranging from 0.89 to 0.99 [24].

### 2.4. Assessment of Instrumental Activities of Daily Living (IADL)

Assessment of IADL was done using the Nottingham extended ADL scale. It is a 22-item scale scored on a 4-point scale from 0 to 1. For example, when a participant indicated “Not at all” as a response to an item on the scale, 0 point was awarded. Other responses to the items on the scale were scored in the same way: “With help”—0 point, “On my own with difficulty”—1 point, and “On my own”—1 point. Maximum score was 22 and minimum score was zero. Higher scores mean greater independence [25]. The Nottingham extended ADL scale has responsiveness of (SRM = 0.9) when compared with the Frenchay Activities Index (SRM = 0.5). The two scales also show good correlations before (*ρ* = 0.8) and after treatment (*ρ* = 0.8) [26].

The IADL raw scores had a mean of 8.77. This mean of IADL raw scores was compared with mean scores of depression, fatigue, and combined depression using unpaired *t*-test. The mean score was used to dichotomize IADL into dependent (score < mean) and independent (score ≥ mean) for the purpose of regression analysis. 

### 2.5. Data Analysis

The data obtained was analysed with inferential statistics of Chi square, unpaired *t*-test, and logistics regression analysis. The descriptive statistics used were mean, median, percentage, and frequencies.

Chi-square was used to find association between dichotomized scores of ADL and those of depression, fatigue, and “combined fatigue and depression.” It was also used to find association between dichotomized scores of ADL and dominant limb paresis. 

Unpaired *t*-test was used to compare the mean scores of IADL with mean scores of depression, fatigue, and combined fatigue and depression. It was also used to compare the mean scores of IADL with mean scores participants with dominant upper limb paresis.

Logistic regression analysis was conducted to control for variation in age and duration of stroke among participants. Level of significance was set at 0.05.

## 3. Results

Among sixty-five stroke patients, 37 (56.9%) males and 28 (43.1%) females participated in the study. Their age ranged between 58 and 80 years and most of them were married 50 (76.9%). Fifty-eight (89.2%) patients were employed and 26 (40%) had tertiary education. Majority 58 (89.2%) were right handed. 

The poststroke characteristics of the individuals show that nine participants (13.6%) had poststroke fatigue alone, 15 participants (23.1%) had poststroke depression alone, 21 participants (32.3%) had both fatigue and depression, and 20 participants (30.77%) did not present with any of fatigue or depression. The total number of fatigue cases recorded was 30 (i.e., 9 + 21) and total number of depression cases recorded was 36 (i.e., 15 + 21) because 21 participants had both fatigue and depression. All these are shown in Table 1.

With the aid of Chi-square analysis, participants with varied poststroke characteristics were compared in dependent and independent ADL. Those with poststroke depression and without poststroke depression were found to be significantly different (*P* = 0.002). Those with poststroke fatigue and without poststroke fatigue were found not to be significantly different (*P* = 0.233), while those with combined fatigue and depression and with no combined fatigue and depression alone were also found to be significantly different (*P* = 0.002). The results in this study reflect that poststroke depression alone and both poststroke fatigue and depression symptoms were significantly associated with functional recovery in ADL (*P* < 0.05), while poststroke fatigue alone was not (*P* > 0.05). These can be observed in Table 2.

In addition, regarding IADL, participants with varied poststroke characteristics were also compared in dependent and independent functional abilities. In this instance, mean IADL scores were used for the comparison. The result showed that participants with poststroke depression and those without poststroke depression were significantly different (*P* = 0.001), while those with poststroke fatigue and those without poststroke fatigue (*P* = 0.2) were not significantly different in functional recovery. However, comparisons of those with both symptoms of poststroke fatigue and poststroke depression and those without poststroke fatigue and poststroke depression were significantly different (*P* = 0.001) in functional recovery. The result indicates that poststroke depression alone and both poststroke fatigue and poststroke depression significantly influenced functional recovery in IADL (*P* < 0.05) while poststroke fatigue alone did not (*P* > 0.05). These are shown in Table 3.

A multivariate analysis was also conducted to control for the influence of differences in age and duration of stroke on functional recovery in ADL and IADL of participants. It was observed that there were no changes in outcome as regards the effect of depression as well as combined depression and fatigue on functional recovery in ADL and IADL before control and after control for the confounders. What is noteworthy, however, was the result of control of these confounders on poststroke fatigue and IADL. An initial odds ratio and 95% confidence interval of 2.51 (0.92–6.84), *P* = 0.073, proceeded toward a higher value of 3.10 (0.99–9.64), *P* = 0.055 (Table 4).

In Table 5, a comparison of stroke participants with dominant and nondominant upper limb paresis on ADL and IADL functional abilities was made. It was observed that dominant upper limb paresis in this study appeared to have no effect on dependent and independent ADL (*P* = 0.67) and IADL (*P* = 0.5).

## 4. Discussion

In this study, poststroke depression alone was significantly negatively related to functional recovery in ADL and IADL. This observation is similar to those of previous studies by researchers such as Lai et al. [8], Nannetti et al. [10], and van de Weg et al. [26]. The probable reason for this observation has been attributed to low quality of life, likely emanating from multifactorial causes such as feelings of hopelessness, helplessness, anxiety, and dehumanization [27, 28] subsequent to stroke. Although this study did not assess those factors, stroke survivors who lack the support of their spouse especially widows and widowers may suffer from hopelessness, helplessness, and anxiety. About a quarter of the elderly females in this study were widows and were unemployed. None of them had access to the National Health Insurance scheme, because the scheme does not cover physiotherapy services, for individuals with stroke. 

In addition, poststroke depression was significantly related to functional recovery in ADL and IADL. This finding is in line with previous reports which suggested that poststroke depression has a negative impact on functional recovery process after discharge [10] and that poststroke depression could also predict functional independence of stroke survivors at discharge [8]. Lai et al. [8] also reported that stroke patients with depression were less likely than those without depression to achieve ADL and attain independence in some IADL. 

A further observation in this study also was that poststroke fatigue alone did not significantly relate to ADL and IADL. This seems to suggest that poststroke fatigue may not independently influence recovery of ADL or IADL. The fact that presence of both poststroke fatigue and poststroke depression in participants in this study significantly influenced functional recovery in ADL and IADL seems to suggest that delay in functional recovery in poststroke individuals with poststroke fatigue could be augmented by the presence of poststroke depression. This probably is the reason why some researchers recommend that, in examining outcomes of poststroke fatigue on functional abilities, confounders such as poststroke depression should be controlled [5, 7].

A notable limitation in this study was that participants' inclusion as regards age and duration of stroke was not standardized. This is expected to affect the individual patient's level of poststroke depression, fatigue, and ADL. This limitation was, however, addressed by controlling for duration of stroke and the effect of this on ADL and IADL functional recovery. Interestingly, however, outcome of effect remained the same in most subgroups of participants except for IADL functional recovery of patients with poststroke fatigue. In this latter subgroup, the odds ratio and 95% confidence interval tended toward a higher value resulting in a less probability value. The implication of this is that probably if the sample size was larger, age and duration of stroke of participants could have had a negative influence on poststroke fatigue and IADL. This area is open to further study. 

Another observation worthy of note in this study is the issue of dominant limb paresis. It is expected that presence of dominant upper limb paresis should influence depression; however, the result of this study appears to be on the contrary. This is because there was no significant relationship between participants with dominant and non-dominant upper limb paresis and functional recovery in ADL and IADL. This result cannot be easily explained.

Furthermore, the cross-sectional survey design used in this study did not permit assessment of poststroke baseline deficits and the degree of change in the deficits with time. This prevented an inference of causality as the degree of recovery of individual participant from onset of stroke to time of participation in the study could not be assessed.

## 5. Conclusion

In this study, participants with poststroke depression have less functional independence in ADL and IADL while functional recovery of those with poststroke fatigue alone seems not to be influenced except when poststroke depression is also present. It is recommended that a longitudinal study with a larger sample size is undertaken so that the result of this study may be more generalized.

## Figures and Tables

**Table 1:** Characteristics of stroke survivors in the study (*N* = 65).

Variables	*n*	%
Sex		
Male	37	56.9
Female	28	43.1
Marital status		
Married	50	76.9
Not married	15	23.1
Educational level		
None	14	21.5
Primary/secondary	25	38.5
Tertiary	26	40.0
Occupation		
Employed	58	89.2
Not employed	7	10.8
Duration of stroke (years)		
<1	26	40.0
1-2	19	29.2
3-4	13	20.0
>4	7	10.8
Upper limb dominance		
Right	58	89.2
Left	7	10.8
Stroke deficit		
Dominant upper limb paresis	22	33.8
Nondominant upper limb paresis	43	66.2
Poststroke characteristic		
Depression alone	15	23.1
Fatigue alone	9	13.8
Combined depression and fatigue	21	32.3
No fatigue and no depression	20	30.8
Activity of daily living status		
Dependent	36	55.4
Independent	29	44.6
Instrumental activity of daily living status		Mean ± SD
Dependent/independent	65	8.77 ± 6.52

**Table 2:** Influence of stroke related variables on activities of daily living (ADL), *N* = 65.

Stroke variable	ADL (dependent)		ADL (independent)		*P*
	*n*	%	*n*	%	
Depression	26	72.2	10	34.5	0.002*
No depression	10	27.8	19	65.5	
Fatigue	19	52.8	11	37.9	0.233*
No fatigue	17	47.2	18	62.1	
Combined fatigue and depression	16	44.4	5	17.2	0.02*
No fatigue no depression and fatigue alone/depression alone*	20	55.6	24	82.8	

*No combined fatigue and depression: no fatigue no depression + fatigue alone/depression alone.

**Table 3:** Influence of stroke related variables on instrumental activities of daily living (IADL), *N* = 65.

Stroke variable	IADL (dependent)		Stroke variable	IADL (independent)		*P*
	*n*	Mean ± SD		*n*	Mean ± SD	
Depression	36	6.4 ± 5.9	No depression	29	11.7 ± 6.1	0.001*
Fatigue	30	7.7 ± 6.6	No fatigue	35	9.7 ± 6.6	0.20
Combined fatigue and depression	21	5.0 ± 4.8	No fatigue, no depression and fatigue alone/depression alone*	44	10.6 ± 6.5	0.001*

*No combined fatigue and depression: no fatigue no depression + fatigue alone/depression alone.

**Table 4:** Multivariate analysis, controlling for influence of differences in age and duration of stroke on outcome.

Poststroke characteristic	ADL		IADL	
	Before control OR (95% CI)	After control OR (95% CI)	Before control OR (95% CI)	After control OR (95% CI)
	*P*	*P*	*P*	*P*
Depression	4.94 (1.72–14.22)	4.72 (1.47–15.17)	6.78 (2.20–20.93)	6.37 (1.85–20.27)
	0.003	0.009	0.001	0.003
Fatigue	1.83 (0.68–4.45)	2.44 (0.78–7.63)	2.51 (0.92–6.84)	3.10 (0.99–9.64)
	0.235	0.126	0.073	0.055
Combined depression and fatigue	6.4 (1.16–35.44)	8.37 (1.05–66.97)	5.36 (1.71–16.74)	6.19 (1.71–22.41)
	0.034	0.045	0.004	0.005

OR: odds ratio; CI: confidence interval, *P*: probability value.

**Table 5:** Influence of dominant upper limb paresis on functional independence (*N* = 65).

	Activity of daily living (ADL)				*P*
Stroke deficit	Dependent		Independent		
	*n*	%	*n*	%	
Dominant limb paresis	13	20.0	9	13.8	0.67
Nondominant limb paresis	23	35.4	20	30.8	

	Instrumental activity of daily living (IADL)				
	*n*	Mean ± SD			

Dominant limb paresis	22	8.0 ± 6.78			0.5
Nondominant limb paresis	43	9.2 ± 6.43			
